# Supplementary material for: Content-rich biological network constructed by mining PubMed abstracts
Source: BMC Bioinformatics. 2004 Oct 8;5:147. doi: 10.1186/1471-2105-5-147 (PMC528731; doi:10.1186/1471-2105-5-147)
Supplement: Additional File 2 — The original results of the above study (non-essential files are deleted to keep the file size under the limit set by BMC bioinformatics). [file 1471-2105-5-147-S2.bz2 › chilibotAdditionalFile2/dip30/721ID11799122E18058/html/ENVZ_OMPR.html]

 


 **ENVZ** and **OMPR** 
  
Found 178 abstracts in PubMed, retrieved 30.  
 

 What does Google say? 
 PDF only 
| .edu only 

---

**Interactive relationship** (e.g. stimulation, inhibition, etc)

**Neutral relationship**- The critical role of DNA in the equilibrium between  **OmpR**  and phosphorylated  **OmpR**  mediated by  **EnvZ**  in Escherichia coli.  Ref: 11158569 Proc Natl Acad Sci U S A, 2001
- Phosphorylation alters the interaction of the response regulator  **OmpR**  with its sensor kinase  **EnvZ** .  Ref: 11799122 J Biol Chem, 2002
- In the present work, we have used fluorescence anisotropy and phosphotransfer assays to examine  **OmpR**  interactions with  **EnvZ** .  Ref: 11799122 J Biol Chem, 2002
- **EnvZ**  can also dephosphorylate phospho  **OmpR**   **OmpR**  P to control the cellular level of  **OmpR**  P.  Ref: 11799122 J Biol Chem, 2002
- Interaction of  **EnvZ** , a sensory histidine kinase, with phosphorylated  **OmpR** , the cognate response regulator.  Ref: 12453215 Mol Microbiol, 2002
- **EnvZ**   **OmpR**  interaction and osmoregulation in Escherichia coli.  Ref: 11973328 J Biol Chem, 2002
- The osmosensor,  **EnvZ** , has dual enzymatic functions with  **OmpR**  kinase and  **OmpR**  P phosphatase.  Ref: 12453214 Mol Microbiol, 2002
- We demonstrated that  **OmpR**  P is able to interact with EnvZc with a similar affinity to  **OmpR**  and serves as an effective substrate for the  **EnvZ**  phosphatase.  Ref: 12453215 Mol Microbiol, 2002
- The classic sensor kinase  **EnvZ**  and acetyl phosphate collaborate to produce the optimum level of  **OmpR**  P needed for autoinduction.  Ref: 12068808 Mol Microbiol, 2002
- The expression of GST fusion proteins carrying the corresponding portions of  **EnvZ**  strongly interfered with the activation of porin gene expression by  **OmpR** .  Ref: 11325944 J Bacteriol, 2001
- **OmpR**  P bound to EnvZc was readily released upon the addition of  **OmpR** , indicating that  **OmpR**  and  **OmpR**  P can compete for the binding to  **EnvZ** .  Ref: 12453214 Mol Microbiol, 2002
- At low osmolarity,  **OmpR**  P levels are low because of either low  **EnvZ**  kinase or high  **EnvZ**  phosphatase activities.  Ref: 11799122 J Biol Chem, 2002
- **EnvZ**  is a bifunctional enzyme that possesses both  **OmpR**  kinase and phosphorylated  **OmpR**   **OmpR**  P phosphatase activities and thus controls the cellular level of  **OmpR**  P.  Ref: 11158569 Proc Natl Acad Sci U S A, 2001
- Although both  **ompR**  and  **envZ**  genes are located on the ompB locus under the control of the ompB promoter and transcribed as a single polycistronic mRNA, the expression of  **envZ**  is known to be significantly less than  **ompR** .  Ref: 11973328 J Biol Chem, 2002
- The model predicts that when  **EnvZ**  is much less abundant than  **OmpR** , as is the case in E. coli, the steady state level of phosphorylated  **OmpR**   **OmpR**  P is insensitive to variations in the concentration of  **EnvZ** .  Ref: 12522261 Proc Natl Acad Sci U S A, 2003
- In an in vitro assay system, the addition of  **OmpR**  to the reaction mixture consisting of the cytoplasmic domain of  **EnvZ**  EnvZc and ATP produces a barely detectable amount of  **OmpR**  P because of the dual activities of  **EnvZ** .  Ref: 11158569 Proc Natl Acad Sci U S A, 2001
- In Escherichia coli, the sensor kinase  **EnvZ**  detects a change in the osmotic environment and phosphorylates the response regulator  **OmpR** .  Ref: 10873450 J Mol Biol, 2000
- pta  **envZ**  double mutants had a residual level of porin transcription, implicating alternative means of  **OmpR**  phosphorylation under nutrient limitation.  Ref: 11700349 Microbiology, 2001
- **EnvZ**  is a sensory histidine kinase in Escherichia coli to regulate the phosphorylation of  **OmpR** , its cognate response regulator, required for the expression of genes for outer membrane porin proteins.  Ref: 12453215 Mol Microbiol, 2002
- Histidine kinase  **EnvZ** , a transmembrane osmotic sensor for Escherichia coli, is a bifunctional enzyme having  **OmpR**  its cognate response regulator kinase and phosphorylated  **OmpR**   **OmpR**  P phosphatase activities.  Ref: 12139613 Mol Microbiol, 2002
- The histidine kinase phosphatase  **EnvZ**  helps Escherichia coli adapt to osmotic shock by controlling the phosphorylation state of the transcription factor  **OmpR** , which regulates the levels of the outer membrane porin proteins OmpF and OmpC.  Ref: 10973966 J Biol Chem, 2000
- **EnvZ** , a histidine kinase phosphatase in Escherichia coli, responds to the osmolarity changes in the medium by regulating the phosphorylation state of the transcription factor  **OmpR** , which controls the expression levels of outer membrane porin proteins OmpF and OmpC.  Ref: 11973328 J Biol Chem, 2002
- We propose that the apparent inhibitory effect of DNA on the  **EnvZ**  phosphatase function is caused by sequestrating  **OmpR**  P from the reaction as a result of  **OmpR**  P binding to DNA.  Ref: 11158569 Proc Natl Acad Sci U S A, 2001

**Non-interactive relationship** (e.g. studied together, co-existance, homology, etc.)

- The SsrB regulon is modulated by the two component regulatory systems PhoP PhoQ and  **OmpR**   **EnvZ** , and is upregulated in the intracellular microenvironment.  Ref: 10844662 Mol Microbiol, 2000
- **EnvZ** , a histidine kinase, and its cognate response regulator  **OmpR**  of Escherichia coli are responsible for adaptation to external osmotic changes by regulating the levels of the outer membrane porin proteins, OmpF and OmpC.  Ref: 12453214 Mol Microbiol, 2002
- Neither trxB nor trxA affected gene regulation mediated by another dual component system tested,  **EnvZ**   **OmpR** .  Ref: 11114904 J Bacteriol, 2001
- Although hilC is regulated by all environmental conditions and regulatory factors that modulate hilA expression, hilC is not required for the regulation of hilA by any conditions or factors except  **EnvZ**   **OmpR** .  Ref: 11292791 J Bacteriol, 2001
- It was observed that during exponential growth in L broth medium there were approximately 3500 and 100 molecules per cell of  **OmpR**  and  **EnvZ** , respectively.  Ref: 11973328 J Biol Chem, 2002
- These findings support the hypothesis that osmotic signals regulate the level of the cellular concentration of  **OmpR**  P by modulating the ratio of kinase to phosphatase activity of the bifunctional enzymatic activities of  **EnvZ** .  Ref: 12453215 Mol Microbiol, 2002
- To test these predictions, we have perturbed the porin regulatory circuit in E. coli by varying the expression levels of  **EnvZ**  and  **OmpR** .  Ref: 12522261 Proc Natl Acad Sci U S A, 2003
- Mutants of the arcB, cyaA, lacZYA,  **ompR**   **envZ** , phnR, pstB, pstCA, pstS, pstSCAB phoU, recA, and torSTRCAD genes or operons were isolated as antibiotic resistant colonies after the introduction into bacteria carrying a Red expression plasmid of synthetic PCR generated DNA.  Ref: 10829079 Proc Natl Acad Sci U S A, 2000
- Under these conditions, the anaeroresponsive ArcB ArcA system, the osmoresponsive  **EnvZ**   **OmpR**  system and the response regulator UvrY showed the most drastic changes.  Ref: 12366850 Mol Microbiol, 2002
- The histidine kinase response regulator system  **EnvZ**   **OmpR**  of Escherichia coli regulates transcription of the genes ompF and ompC, encoding two porins of the outer membrane.  Ref: 11533042 J Biol Chem, 2001
- **OmpR**  and  **EnvZ**  comprise a two component system that regulates the porin genes ompF and ompC in response to changes in osmolarity.  Ref: 11799122 J Biol Chem, 2002
- Escherichia coli modulates its porin expression through a histidine kinase,  **EnvZ** , and its cognate response regulator,  **OmpR** .  Ref: 11158569 Proc Natl Acad Sci U S A, 2001
- A variety of genes hilC, hilD, fis, sirA barA, csrAB, phoB, fadD,  **envZ**   **ompR** , fliZ, hilE, ams, lon, pag, and hha have been identified that exert positive or negative effects on hilA expression, although the mechanisms by which these gene products function remain relatively unclear.  Ref: 12595445 Infect Immun, 2003
- Formation of the stoichiometric complex of  **EnvZ** , a histidine kinase, with its response regulator,  **OmpR** .  Ref: 12453214 Mol Microbiol, 2002
- **EnvZ**  is autophosphorylated by intracellular ATP on a histidine residue, and it transfers the phosphoryl group to an aspartic acid residue of  **OmpR** .  Ref: 11799122 J Biol Chem, 2002
- The  **EnvZ**   **OmpR**  system in Escherichia coli, which regulates the expression of the porins OmpF and OmpC, is one of the simplest and best characterized examples of two component signaling.  Ref: 12522261 Proc Natl Acad Sci U S A, 2003
- RESULTS Under standard aerobic growth conditions, it is well known that the osmoregulatory profile of the outer membrane porins OmpC and OmpF is mainly regulated by the  **EnvZ**   **OmpR**  phosphorelay system in response to medium osmolarity.  Ref: 10947842 Genes Cells, 2000
- Neither the transfer of the phosphoryl group from  **EnvZ**  approximately P to  **OmpR**  nor the  **EnvZ**  mediated  **OmpR**  approximately P dephosphorylation were affected by one of the tested solutes.  Ref: 11533042 J Biol Chem, 2001
- The influence of cyaA, rpoS, ackA and pta, as well as the two component  **envZ**   **ompR**  system, was studied under glucose and N limitation in chemostat cultures.  Ref: 11700349 Microbiology, 2001
- **EnvZ**  and  **OmpR**  are a transmembrane sensor and its cognate response regulator, respectively, regulating the transcription of porin genes in response to medium osmolarity in Escherichia coli.  Ref: 12754242 J Bacteriol, 2003
- In alpha proteobacteria, HPRK is located downstream of genes encoding a two component system of the  **EnvZ**   **OmpR**  family.  Ref: 12867744 J Mol Microbiol Biotechnol, 2003
- CONCLUSIONS Under anaerobic growth conditions, porin expression is tuned not only by the authentic osmo resposive  **EnvZ**  sensor, but also by the anaero responsive ArcB sensor, in an  **OmpR**  dependent manner.  Ref: 10947842 Genes Cells, 2000
- Among the four reactions occurring in the mixture, only the  **EnvZ**  phosphatase activity was inhibited severely by the specific DNA, in contrast to the previous report by Kenney and her associates that DNA stimulates  **OmpR**  phosphorylation by  **EnvZ**  Ames, S.  Ref: 11158569 Proc Natl Acad Sci U S A, 2001
- The operon flhDC is controlled by numerous regulators including H NS, CRP,  **EnvZ**   **OmpR** , QseBC and LrhA.  Ref: 12864862 Mol Microbiol, 2003
- The levels of  **OmpR**  and  **EnvZ**  in MC4100 cells grown in a high osmolarity medium nutrient broth with 20% sucrose were about the same as those grown in L broth.  Ref: 11973328 J Biol Chem, 2002
- Using these strains we have shown that, consistent with the predictions of our model, the transcription of ompC and ompF is indeed robust or insensitive to a wide range of expression levels of both  **EnvZ**  and  **OmpR** .  Ref: 12522261 Proc Natl Acad Sci U S A, 2003
- The phosphoryl group of H6 HepK is transferred rapidly and efficiently to both H6DevR and H6DevRA but not to His tagged  **OmpR** , whose cognate sensor kinase is  **EnvZ** .  Ref: 12637541 J Biol Chem, 2003
- Partial sequence analysis of a large native plasmid approximately 120 kb, designated pA5H5, from a highly virulent Australian isolate revealed a putative two component regulatory system with inferred identity to the  **OmpR**  family of two component transcriptional regulatory proteins and  **EnvZ**  sensor kinases.  Ref: 12398213 Int J Med Microbiol, 2002
- Here we examined the levels of  **EnvZ**  and  **OmpR**  in the wild type strain MC4100 by quantitative Western blot analysis using anti  **OmpR**  and anti EnvZc cytoplasmic domain of  **EnvZ**  antisera.  Ref: 11973328 J Biol Chem, 2002
- The Escherichia coli  **OmpR**   **EnvZ**  two component regulatory system, which senses environmental osmolarity, also regulates biofilm formation.  Ref: 11717281 J Bacteriol, 2001
- Here, we demonstrate that the cytoplasmic kinase domain of  **EnvZ**  EnvZc and  **OmpR**  are able to form a 1 1 complex detected by native PAGE.  Ref: 12453214 Mol Microbiol, 2002
- Porin production was minimally dependent on  **EnvZ**  under N limitation due to  **OmpR**  phosphorylation by acetyl phosphate.  Ref: 11700349 Microbiology, 2001
- Furthermore, we establish that the antibiotic resistance phenotype is primarily due to the induction of the MarRAB regulatory system by the AOAs, while other regulatory pathways that also converge into micF modulation  **OmpR**   **EnvZ** , SoxRS, and Lrp remained unaltered.  Ref: 11353631 Antimicrob Agents Chemother, 2001
- Here, we show that FadD, FliZ, PhoB, and  **EnvZ**   **OmpR**  also require the URS to regulate hilA.  Ref: 11292791 J Bacteriol, 2001
- We speculate that  **EnvZ**   **OmpR**  regulates hilA by altering the expression and or activity of hilC, while all other regulatory conditions and mutations regulate hilA by modulating hilD posttranscriptionally.  Ref: 11292791 J Bacteriol, 2001
- In Escherichia coli, the two component regulatory system that controls the expression of outer membrane porins in response to environmental osmolarity consists of the sensor kinase  **EnvZ**  and the response regulator  **OmpR** .  Ref: 11812125 J Mol Biol, 2002
- The  **EnvZ**   **OmpR**  histidyl aspartyl phosphorelay HAP system in Escherichia coli regulates the expression of ompF and ompC, the major outer membrane porin genes, in response to environmental osmolarity changes.  Ref: 12767831 J Mol Biol, 2003
- However, to date no accurate estimation for the amounts of  **EnvZ**  and  **OmpR**  in the cell has been carried out.  Ref: 11973328 J Biol Chem, 2002
- We have analyzed a mathematical model of the  **EnvZ**  mediated cycle of  **OmpR**  phosphorylation and dephosphorylation.  Ref: 12522261 Proc Natl Acad Sci U S A, 2003
- Here, we re examined the recent paper Mattison and Kenney, in which the authors reported that phosphorylated  **OmpR**   **OmpR**  P is unable to bind to  **EnvZ** , thus casting doubts on the role of the  **EnvZ**  phosphatase activity in vivo.  Ref: 12453215 Mol Microbiol, 2002
